# Identification of hub pathways and drug candidates in gastric cancer through systems biology

**DOI:** 10.1038/s41598-022-13052-0

**Published:** 2022-06-01

**Authors:** Seyed Reza Salarikia, Mohammad Kashkooli, Mohammad Javad Taghipour, Mahdi Malekpour, Manica Negahdaripour

**Affiliations:** 1 Student Research Committee, Shiraz University of Medical Sciences, Shiraz, Iran; 2 Pharmaceutical Sciences Research Center, Shiraz University of Medical Science, Shiraz, Iran; 3 Department of Pharmaceutical Biotechnology, School of Pharmacy, Shiraz University of Medical Sciences, P.O. Box 71345-1583, Shiraz, Iran

**Keywords:** Computational biology and bioinformatics, Gastroenterology

## Abstract

Gastric cancer is the fourth cause of cancer death globally, and gastric adenocarcinoma is its most common type. Efforts for the treatment of gastric cancer have increased its median survival rate by only seven months. Due to the relatively low response of gastric cancer to surgery and adjuvant therapy, as well as the complex role of risk factors in its incidences, such as protein-pomp inhibitors (PPIs) and viral and bacterial infections, we aimed to study the pathological pathways involved in gastric cancer development and investigate possible medications by systems biology and bioinformatics tools. In this study, the protein–protein interaction network was analyzed based on microarray data, and possible effective compounds were discovered. Non-coding RNA versus coding RNA interaction network and gene-disease network were also reconstructed to better understand the underlying mechanisms. It was found that compounds such as amiloride, imatinib, omeprazole, troglitazone, pantoprazole, and fostamatinib might be effective in gastric cancer treatment. In a gene-disease network, it was indicated that diseases such as liver carcinoma, breast carcinoma, liver fibrosis, prostate cancer, ovarian carcinoma, and lung cancer were correlated with gastric adenocarcinoma through specific genes, including *hgf*, *mt2a*, *mmp2*, *fbn1*, *col1a1*, and *col1a2.* It was shown that signaling pathways such as cell cycle, cell division, and extracellular matrix organization were overexpressed, while digestion and ion transport pathways were underexpressed. Based on a multilevel systems biology analysis, hub genes in gastric adenocarcinoma showed participation in the pathways such as focal adhesion, platelet activation, gastric acid secretion, HPV infection, and cell cycle. PPIs are hypothesized to have a therapeutic effect on patients with gastric cancer. Fostamatinib seems a potential therapeutic drug in gastric cancer due to its inhibitory effect on two survival genes. However, these findings should be confirmed through experimental investigations.

## Introduction

Gastric cancer was responsible for nearly one million new cancer cases in 2020, ranking the fifth among all cancers in regard to incidence. It was the fourth cause of cancer death, with approximately 769,000 deaths in 2020^1^. Gastric adenocarcinoma, the most common type of gastric cancer, originates from stomach mucosal epithelium and accounts for nearly 90% of all gastric cancer cases^2^. Gastric cancer incidence varies in different parts of the world. The highest incidence rate of gastric cancer is found in Central and East Asia as well as Latin America, with the first rank belonging to South Korea with an average incidence rate of 60 per 100,000 for men and 25 per 100,000 for women. North and East Africa have the lowest incidence rate^3^.

There are different risk factors for gastric cancer, including *Helicobacter pylori*, cigarette smoking, alcohol, dietary salt, food preservation, and genetic syndromes^4^, among them *H. pylori* is the most salient one. According to recent studies, the risk of gastric cancer decreased in patients undergoing *H. pylori* eradication therapy (pooled incidence rate ratio of 0.53, 95% CI 0.44–0.64)^5^. Beyond that, genetic factors have a profound impact on gastric cancer; it seems that E-cadherin (*cdh1*) mutation leads to a specific type of gastric cancer called hereditary diffuse gastric cancer^6^. Some viruses such as Epstein-Barr virus (EBV) and human papilloma virus (HPV) may also induce gastric cancer. EBV has been found in nearly 9% of patients with gastric cancer^7^, but HPV prevalence is widely different. While the HPV DNA detection in the tumor cells of gastric cancers was not confirmed in Central Europe^8^, in two studies performed in China, the HPV DNA was detected in 29% and 47.5% of cases^9,10^. Gastric cancer survival rate also varies in different parts of the world; in some countries such as the UK, New Zealand, Australia, Canada, Denmark, and Norway, the survival rate is lower than 40%, while it is higher in the high-incidence countries including South Korea and Japan. The probable reason is that the two latter countries have an appropriate plan for screening patients by using radiography and endoscopy, which may cause the survival rate to increase up to 70%^11^.

A multidisciplinary approach is essential for the treatment of gastric cancer, which should be decided by a team including at least one surgeon, a pathologist, a gastroenterologist, as well as medical and radiation oncologists^12^. In case of surgical intervention, which is the gold standard treatment for gastric cancer^13^, complete surgical resection with suitable lymphadenectomy and endoscopic mucosal resection (EMR) can be appropriate to treat early gastric cancer^14^. Furthermore, adjuvant and neoadjuvant therapy, including chemotherapy and radiotherapy, are very effective in treating gastric cancer. This treatment strategy, which is widely used in some areas of the world, such as Asia and the United States, can reduce tumor size or its invasive behaviors. It may also improve the efficacy of surgical resection as the main way of gastric cancer treatment and could even reduce the side effects after surgery^15^. In this regard, pre-operative chemotherapy with epirubicin, cisplatin, and infused fluorouracil (ECF) regimen could significantly decrease tumor size and increase the patient overall survival rate^16^.

Although there is still doubt about the effectiveness of gastric cancer drugs, several molecules such as bemarituzumab, which targets proto-oncogene c-SRC (*src*), tyrosine-protein kinase (*ptk*), and Mastinib, which targets fibroblast growth factor 2 (*fgf2*), may be the potential drugs under investigation in clinical trials^17^. Several pathways have a substantial role in gastric cancer development, such as the vascular endothelial growth factor (VEGF) pathway, phosphatidylinositol 3-kinase (PI3K)/ AKT/ mammalian target of rapamycin (mTOR) signaling pathway, hepatocyte growth factor (HGF)/ tyrosine-protein kinase Met (MET) signaling pathway, Janus kinase (JAK)/ signal transducer and activator of transcription proteins (STAT) signaling pathway, and Wnt signaling pathway^18^. Thus, studying these pathways could help in finding appropriate targets and discovering suitable drugs to improve patients’ survival^19^.

Proton-pomp inhibitor (PPI) drugs reduce acid secretion by blocking the gastric H^+^, K^+^-ATPase. They can be administered in some conditions such as gastro-esophageal reflux^19^. It is noted that PPIs could have dual roles in gastric cancer; some researchers believe that the long-term use of PPIs can increase the risk of gastric cancer, while others claim that they can support chemotherapy efficacy in gastric cancer treatment^20^. This dual role can be the subject of discussion and further study, which can be done through a systemic approach.

Systems biology is a popular way to understand the complexity of systems. It employs computational modeling to better understand the biological processes and analyze them^21^. There are some studies using this approach for finding biomarkers, differentially expressed genes, and important pathways in gastric cancer. For instance, Echizen et al. find out the crucial role of NOX1/ROS signaling pathway in gastric cancer tumorigenesis^22^. In another study by Vizeacoumar and colleagues, candidates for potential biomarkers were discovered including CST1, INHBA, STMN1^23^. He et al. also found potential immune-related prognostic biomarkers of gastric cancer including INHBA, ANGPTL1, ACKR1, GHR^24^. These studies can show the wide perspective of this approach for a better understanding of human diseases.

In this study, systems biology approaches are used to find hub (the most important nodes in the networks) genes in gastric adenocarcinoma, study the related important pathways, and finally suggest potential drugs for treating gastric adenocarcinoma.

## Methods

**Figure 1:**
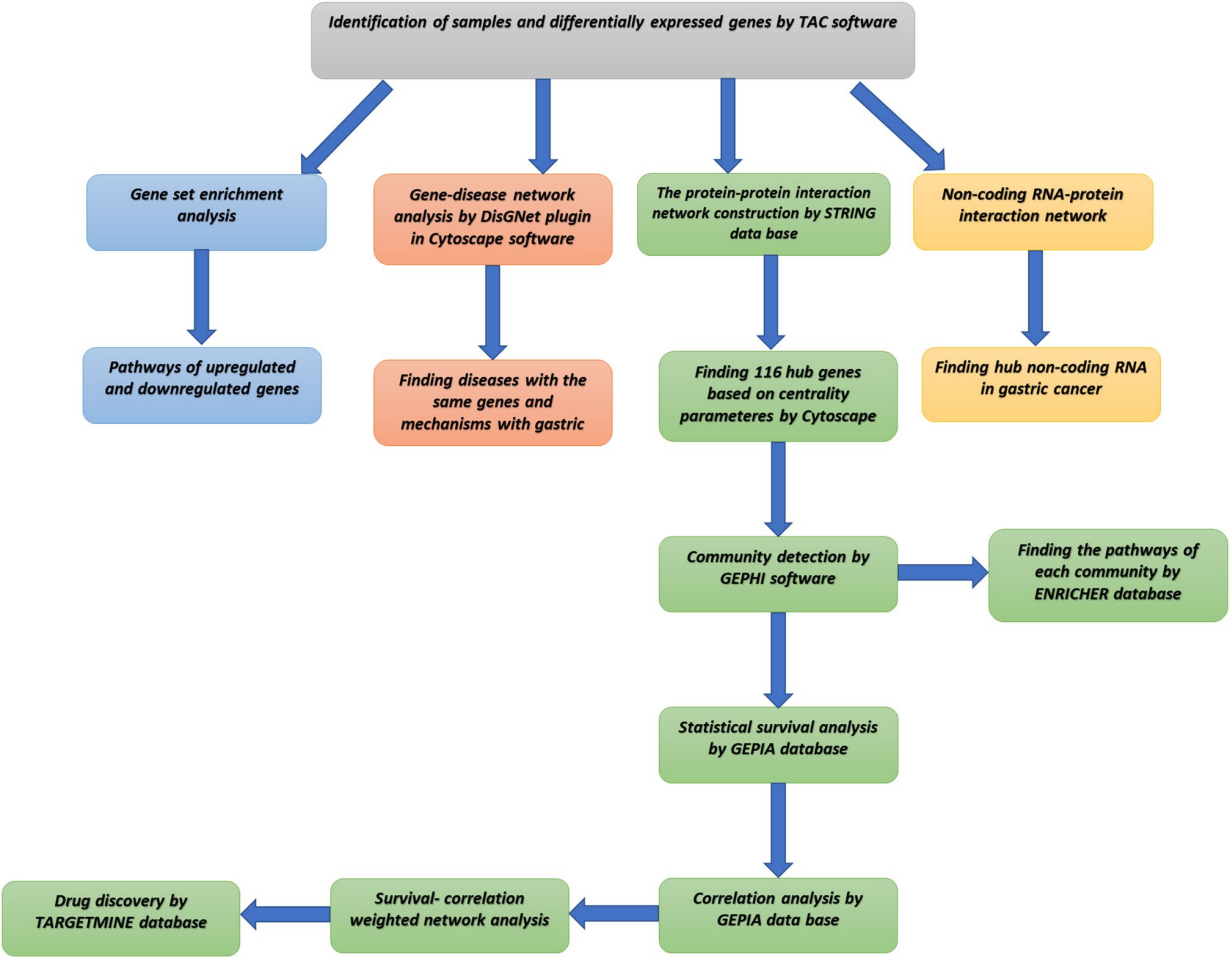
Method diagram showing the summary of applied steps in this study.

The approach used in this study is as follows in brief: gastric cancer was modeled by network analysis tools based on gene expression data, and potential compounds that can affect patients’ survival were detected. Besides, non-coding RNA interactions, viral causes, and pathways were analyzed to better understand the pathological mechanisms of gastric cancer development. A summary of the method used is shown in Fig. 1.

### Identification of samples and differentially expressed genes in gastric cancer

The gene expression omnibus (GEO) database (https://www.ncbi.nlm.nih.gov/geo) was used to download a microarray dataset related to gastric cancer. This database provides a great number of microarrays, RNA seq, and other data freely, so it can be a very useful database for working in the genetics and bioinformatics field. In the advanced search section, all microarray profiles with “gastric cancer” and “u133” keywords in their title or abstract were searched. Forty-five studies about *Homo sapiens* were found. Among them, GSE79973 consists of 10 pairs of gastric cancer tissue and adjacent non-tumor samples with HG-U133_Plus_2 platforms (a single array representing 14,500 well-characterized human genes, which can be used to explore human biology and disease processes), and was selected for further analyses^25^. The samples were analyzed by using transcriptome analysis console (TAC) software with the help of affymetrics U133-plus2 library to identify differentially expressed genes (DEGS). RMA algorithm was applied for data normalization. Then, PCA was used to reduce the dimensions of the dataset, so it could be evaluated if normal versus tumor was separated well. Comparison analysis (t-test) was also done on the dataset to identify differentially expressed genes with a significant *p*-value (*p*-value ≤ 0.05). Finally, gene set enrichment analysis helped to discover the possible related pathways associated with each group of genes. Two outlier samples were excluded. After that in PCA, normal and tumor samples were well separated. Finally, for further analysis, DEGS were detected based on their fold changes (FC ≥ 2.3 or FC ≤ -2.3) at a significance level of (*p*-value ≤ 0.05) (Table S1).

### Gene set enrichment analysis

DEGs were imported in STRING v11.0 (https://string-db.org/)^26^ and ranked based on their fold change (proteins with value/rank section). The STRING database can help to find the relation between different proteins in different species, including *homo sapiens*. Enrichment analysis was done using the gene ontology (GO) database (http://geneontology.org/), which has a good potential for finding different pathways wherein a group of genes has a role. Pathways were filtered based on the false discovery rate (FDR ≤ 0.05).

### The protein–protein interaction network construction

The STRING v11.0 database was employed to construct the protein–protein interaction network of DEGs, and Cytoscape v3.7.2 software was used for visualization and network parameter calculation^27^. Centrality parameters including degree, betweenness, and closeness were calculated for the identification of hub genes in the network using Gephi. Gephi is a powerful software for the visualization and analysis of networks^28^. It was employed for the identification of smaller communities that a group of hub genes form, which are called modules, among 116 selected hub genes. Gephi uses Louvain method for modularity analysis, which is a popular method in community identification^29^. As the last step, two databases of KEGG and gene ontology (GO) at https://maayanlab.cloud/Enrichr/ were used to show the related biological pathways of each module^30–32^. These two databases are good online tools for the detection of related pathways to a group of genes. The upregulated and downregulated hub genes were imported separately to ENRICHR to find the upregulated and downregulated pathways. ENRICHR is a professional online database, which can link a group of genes to other databases, showing the related pathways, diseases, drugs, and other data^33^.

### Non-coding RNA–protein interaction network construction

In this study, non-coding RNA network construction was considered to get a better view on the pathogenesis of gastric cancer. Non-coding RNA expression data were separated from DEGs at the significance level of (*p*-value ≤ 0.05, FC ≥ 2.3 or FC ≤ -2.3). The non-coding versus coding RNA network was constructed using the RNA Interactome Database (https://www.rna-society.org/rnainter/)^34^. A python script was also employed. The network was constructed between 116 and 37 coding hub and non-coding genes, respectively. STRING v11.0 protein–protein interaction network was also added to obtain a complete interaction network.

### Gene-disease network

The 116 hub genes were exported to ENRICHR, and their correlated diseases were extracted from the DisGNet plugin to identify the best matched diseases with these hub genes, based on the *p*-value. The DisGNet plugin in Cytoscape was then used to find the interaction of our 116 hub genes with diseases and their involvement in several diseases^35^. The collected data can suggest the genes that might have role in the metastasis of gastric cancer or other related cancers based on the networks.

### Statistical survival analysis

The GEPIA database (http://gepia.cancer-pku.cn/) was employed for survival analysis^36^. GEPIA is an online tool that can be used for survival, correlation analysis, and gene expression analysis in different types of cancer and normal tissues. We hypothesized survival analysis can discover more reliable drug targets among our hub genes, because it can show the patient outcome in association with the underexpression or overexpression of genes, so a gene with both high centrality parameters in graph and significant impact on the patient survival at the same time might have stronger and more valid evidence for tumor growth progression or inhibition.

The GEPIA uses TCGA and GTEx project data to perform overall survival or disease-free survival analysis using log-rank t-test with adjustable thresholds. It can also calculate the cox proportional hazard ratio and shows 95% confidence interval on survival plots. Log-rank < 0.05 was assumed as the significance level, and the threshold was set on 50 percent.

All the survival plots of 116 hub genes were checked, and their cox proportional hazard ratio was calculated to find the hub genes that had a significant effect on the survival rate. The survival gene expression between tumor and normal data in GEPIA were also checked, and the survival genes without significance differential expression based on GEPIA analyses were excluded.

### Correlation analysis

GEPIA was also used to identify the linear correlation between the discovered survival genes and the rest of hubs. Pearson method was used, and the significance level was set on “R coefficient ≥ 0.5, *p*-value ≤ 0.05” to discover the linear correlation. Again, the genes without significant expression in the tumor and normal samples were excluded based on the GEPIA analysis. The pathways in which a survival gene and its correlations were involved, were found using KEGG and GO in the ENRICHR database. It allowed to identify all possible drug targets with a high effect on tumor growth in order to use them in the experiments for validation.

### Survival-correlation weighted network analysis

To have a better analysis that can be attributed to all gastric cancer populations, the box plot of the GEPIA database was used. In our study, an interaction network was constructed between survivals and their correlations to understand the hypothetical mechanisms that result in poor prognosis in gastric cancer. The weight of edges in this network was the correlation coefficient retrieved from GEPIA.

### Drug discovery

The Targetmine database (https://targetmine.mizuguchilab.org/) was used to find drugs and compounds that have interactions with genes/proteins^37^. Targetmine can search multiple genes in Drug bank and other databases at the same time and show the results. Based on the aforementioned database, the survival genes whose overexpression resulted in better patient survival rates could be targeted with cognate agonists. In this study, to discover any possible synergistic effects of drugs, the genes that had a positive linear correlation with survivals needing agonists were detected. Antagonists were selected against the genes whose underexpression either was related with better survival rates or had a positive linear correlation with this type of survival genes.

### Ethical considerations

The human gene data obtained in the study was derived from a publicly available repository. All experiments were conducted in accordance with relevant guidelines and regulations in the main manuscript.

## Results

### Differentially expressed genes (DEGs) analysis

**Figure 2:**
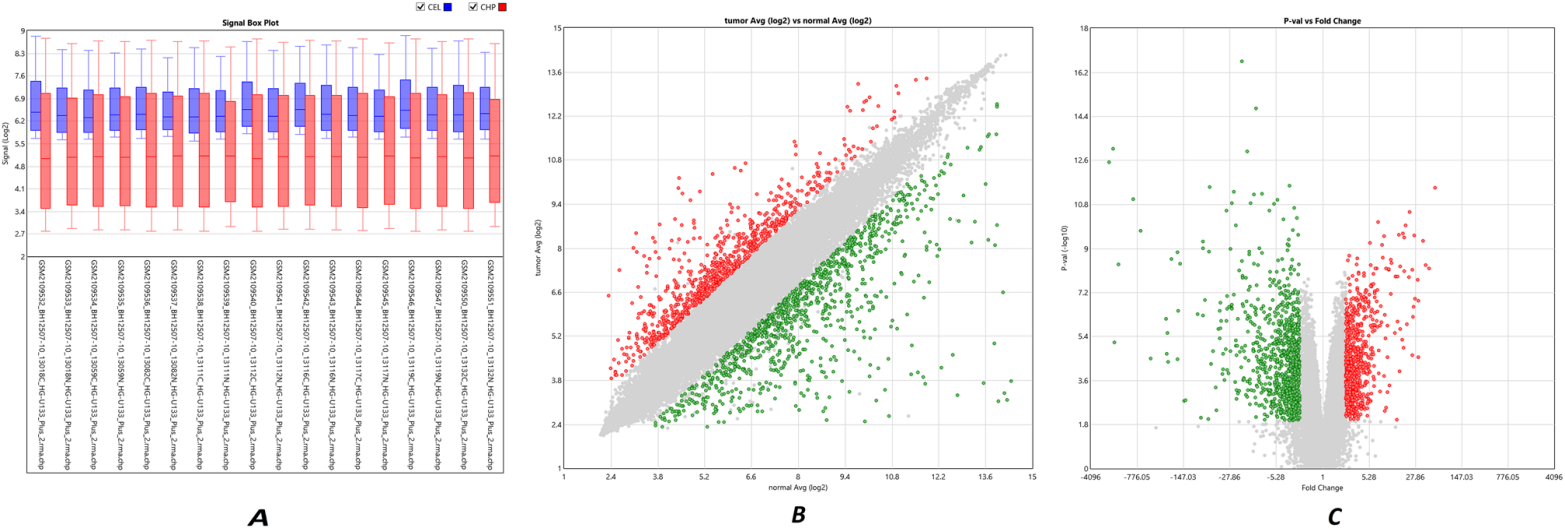
Normalization and gene filtering of the microarray data. (**A**) Box plot gene expression distribution in each patient and data normalization of microarray samples. For each microarray sample, blue box plot (CEL) shows microarray gene expression distribution before normalization, and red box plot (CHP) represents gene expression distibution after normalization. (**B**) The scatter plot of DEGs (differentially expressed genes). It shows varience of the average gene expression in normal versus tumor patients. (**C**) Volcano plot of DEGs (differentially expressed genes) between gastric adenocarcinoma samples versus paired normal tissue. It shows signficant gene expression based on ther *p*-value and fold change. *Note:* In (**B**) and (**C**), red and green dots show upregulated and downregulated genes, respectively. Besides, gray nodes are the DEGS whose fold change is below the determined limit in the study.

The normalized data of gastric adenocarcinoma samples versus paired normal tissue and the box plot gene expression distribution in each patient are shown in Fig. 2A. The microarray gene expression distribution before normalization is shown in blue boxes (CEL). A CEL file is a data file created by Affymetrix DNA microarray image analysis software. It contains the data extracted from "probes" on an Affymetrix GeneChip and can store thousands of data points, which may make the file size large. CEL files can be processed by software algorithms and visualized on a 2D grid as part of an overall genome experiment.

The gene expression distribution after normalization is represented in red boxes (CHP). CHP is a file format generated by Transcriptome analysis console (TAC) after normalization of the expression data by RMA, MSA5 or DABG algorithms. It saves algorithm parameters and summary statistics.

**Figure 3:**
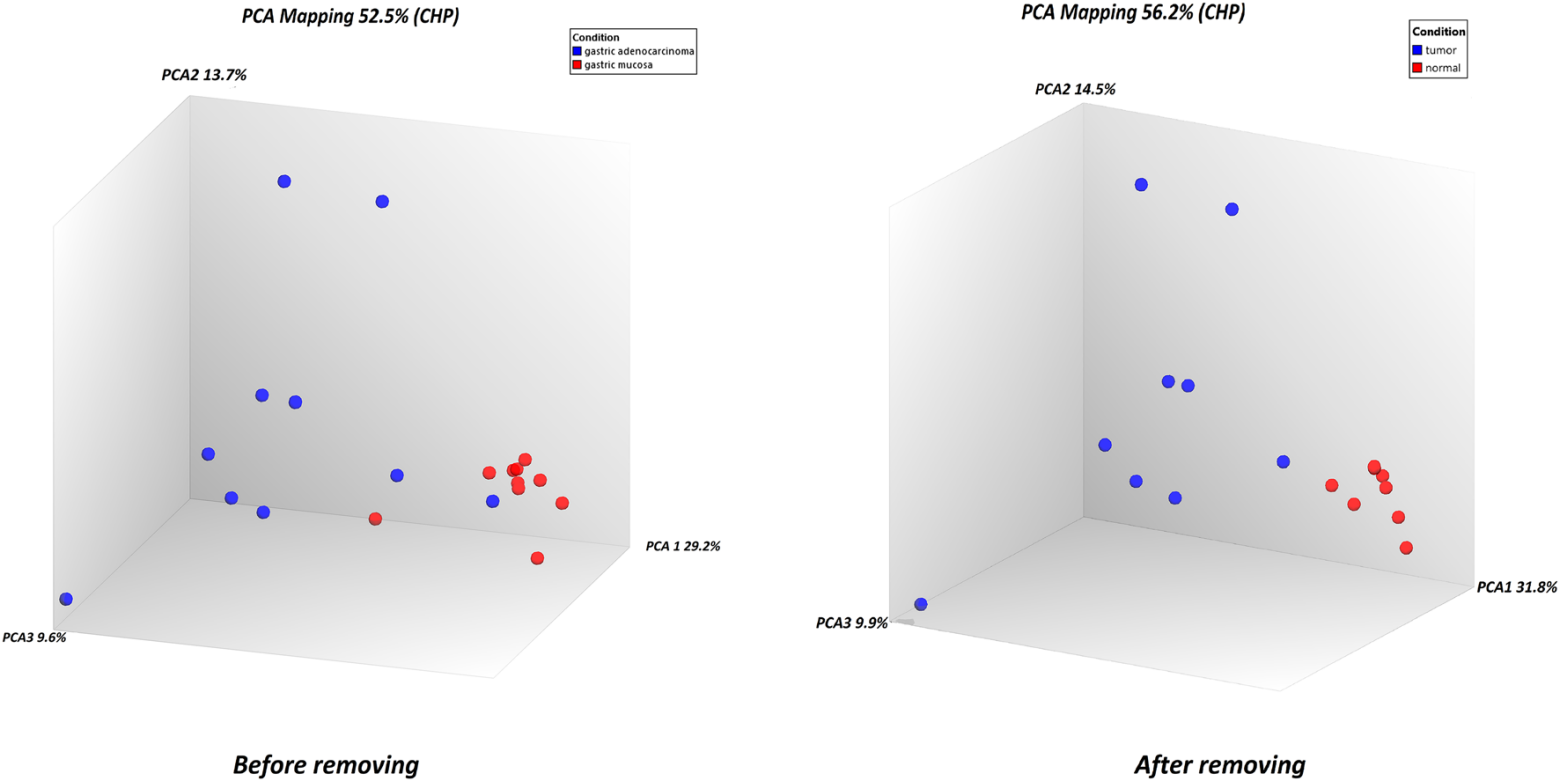
The PCA graph of gastric adenocarcinoma samples versus paired normal tissues before and after removing two outlier samples. Normal patients and tumor patintes are well separated on principal component 1 (PCA1). *Note:* Red dots are normal samples, and blue dots are tumor tissue samples.

According to the PCA results, two samples, including GSM2109548_BH12507-10_13130C_HG-U133_Plus_2.CEL and GSM2109549_BH12507-10_13130N_HG-U133_Plus_2.CEL, were excluded, and others entered further analysis (Fig. 3). DEGs were identified at a significance level of *p*-value ≤ 0.05, FC ≤ − 2.3 or FC ≥ 2.3 (Fig. 2B, C). There were 1768 DEGs, among which 775 genes were overexpressed, and 993 genes were downregulated (Supplementary Table S1).

### Gene set enrichment analysis

The DEGs were ranked based on their fold change and were imported to STRING v11.0 for gene set enrichment analysis based on the GO biological pathways. Filtering of pathways based on false discovery rate (FDR ≤ 0.05) showed the involvement of the overexpressed genes in the extracellular matrix organization, while the downregulated genes were found to play a role in ion transportation and digestion (Supplementary Table S2).

### Protein–protein interaction network analysis

**Figure 4:**
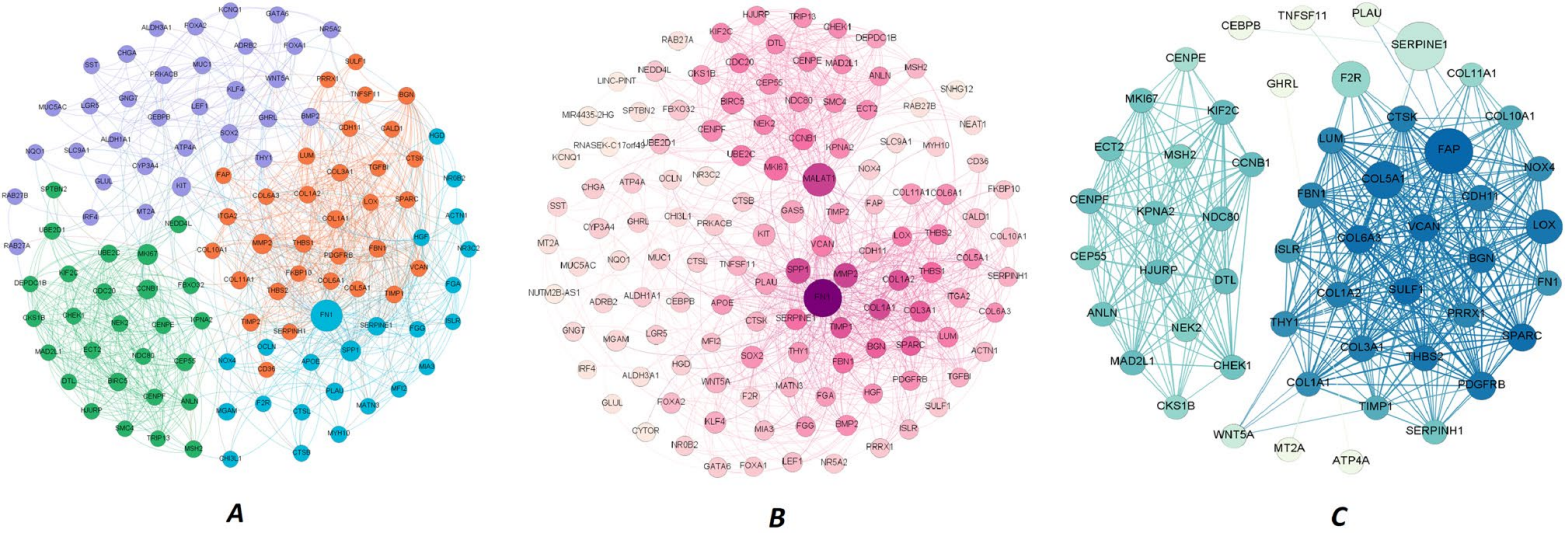
(**A**) 116 hub genes (the most important nodes in the network) and their interaction in the network. Node size represents betweenness, and node color shows modularity. Purple nodes: module 0*;* Green nodes: module1; Orange nodes: module 2; Blue nodes: module 3. (**B**) Hub genes and their correlating microRNA networks in gastric cancer. It shows the effect of microRNA in the hub genes network. The node size demonstrates the degree, while the node color represents betweenness. (**C**) Weighted network analysis between the survival genes and their correlations, which shows separation between agonist and antagonist requiring targets. Node size represents degree, and node color shows betweenness. Edge thickness shows the correlation of nodes.

Fibronectin 1 *(fn1),* collagen type I alpha 1 chain *(col1a1),* matrix metallopeptidase 2 *(mmp2),* collagen type I alpha 2 chain *(col1a2),* secreted protein acidic and cysteine-rich *(sparc),* biglycan *(bgn)* were the first five hub genes based on eigenvector. These genes possibly can control most of the feedback loops involved in tumor cell survival, and interventions against their function may result in dramatic changes in the gastric cancer tumor cells’ growth. The first 116 hub genes had four modules, and module enrichment analysis based on the KEGG pathways showed the involvement of these modules in pathways such as cell cycle, focal adhesion, platelet activation, and gastric acid secretion. It also suggested the HPV infection pathway, which was nearly correlated with extracellular matrix organization. All these pathways were upregulated except gastric acid secretion. Targeting these genes may result in numerous side effects, so we decided to target the hub genes that specifically affected gastric cancer patients’ survival (Supplementary Table S3, Fig. 4A).

### Survival analysis

The survival analysis performed on the overexpressed and underexpressed hub genes represented the significant role of hub genes in the survival of patients suffering from gastric cancer. The overexpression of some hub genes such as *fn1,* platelet-derived growth factor receptor beta (*pdgfrb*), *col1a1*, versican *(vcan),* plasminogen activator inhibitor-1 *(serpine1),* immunoglobulin superfamily containing leucine rich repeat *(islr),* cluster of differentiation 36 *(cd36),* fibrinogen gamma chain *(fgg),* ghrelin and obestatin prepropeptide *(ghrl)*, and coagulation factor II thrombin receptor *(f2r)* showed a negative impact on the survival of patients suffering from gastric cancer. In this group, all of the genes except *cd36*, *ghrl*, and kit proto-oncogene *(kit)* displayed overexpression in gastric cancer, which might suggest their oncogenic effect. On the other hand, the overexpression of some hub genes such as centrosomal protein 55 (*cep55*), centrosome-associated protein E (*cenpe*)*,* and epithelial cell transforming 2 *(ect2)* indicated a positive effect on the survival of gastric cancer cases. Moreover, these genes were overexpressed in the tumor tissue, which might show their role in tumor growth inhibition. Checking the hub genes in GEPIA revealed that they all had significant roles in the prognosis of gastric cancer.

### Correlation analysis

It is speculated that the linear correlated expression between the hub genes might have a synergistic effect on tumor progression, especially if the correlation between non-survival and survival hub genes is of a casual type. Although most of the genes in the network with high centrality parameters were not survival-affecting genes, they had a linear correlation with the survival genes. Thus, they might have a causal effect on the expression of the survival-affecting hub genes. However, it must be investigated in an experimental approach. Our results showed that some genes namely *col3a6*, *pdgfrb, col1a2, fn1, thbs2, sulf1,* and *vcan* had a positive linear correlation with *col1a1* gene. Genes *col1a2*, *col6a3*, *thbs2*, fibroblast activation protein *(fap), sulf1,* and *bgn* showed a positive linear correlation with *vcan* gene, while genes *col1a2, wnt5a, col6a3, thbs1, thy1, vcan, sparc*, and *bgn* indicated a positive linear correlation with *pdgfrb* gene. Genes *mki67*, *anln, ccnb1, cenpf, chek1, dtl*, and *ndc80* displayed a positive linear correlation with *cep55* gene.

It was also found that the genes with a significant role in survival were correlated with each other. Several paired genes including (*f2r, islr), (fn1, col1a1), (islr, col1a1), (islr, f2r), (pdgfrb, col1a1), (pdgfrb, f2r),* and (*vcan, col1a1)* showed a positive correlation with each other, so that using antagonists against both of them could have a synergistic effect on the gastric cancer survival. Moreover, as mentioned above, the overexpression of two other paired genes, *i.e.* (*cep55, cenpe*) and *(cep55, ect2)*, revealed a good impact on survival, so that using their agonist may have a synergistic effect on gastric cancer survival. Interestingly, in this analysis, *cep55* overexpression was found to relate to a positive prognosis in gastric cancer patients.

### Non-coding RNA–protein interaction network construction

Using the complete constructed non-coding RNA protein interaction network, network centrality parameters were calculated for both hub coding and non-coding genes using Gephi. *Fn1,* growth arrest-specific transcript 5 (*gas5*), and metastasis-associated lung adenocarcinoma transcript 1 (*malat1*) were detected as hubs based on degree and betweenness in the network. It showed the central role of *fn1* and *malat1* in the gastric cancer pathogenesis (Fig. 4B).

### Gene-disease network

**Figure 5:**
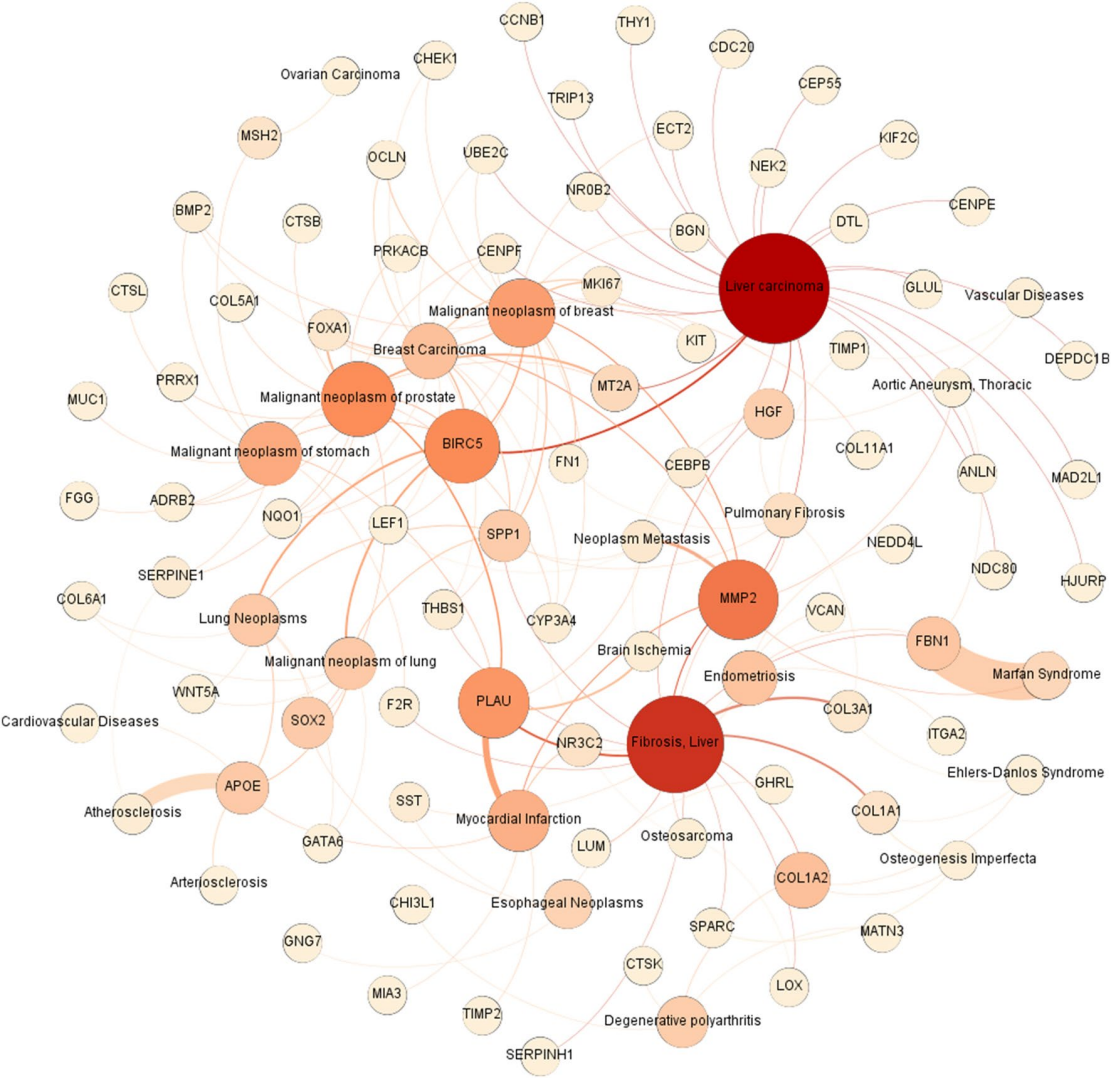
Gene-disease network. It shows not only the diseases and syndromes that have common genes and pathways with gastric adenocarcinoma, but also the common genes participating in different diseases and syndromes. Node size represents the degree (the bigger size shows the higher degree). The node color exhibits betweenness (the darker color indicates the higher betweenness).

It was found that 116 hub genes were also involved in liver carcinoma, breast carcinoma, liver fibrosis, prostate cancer, ovarian carcinoma, lung cancer, brain ischemia, atherosclerosis, arteriosclerosis, aortic aneurysm, cardiovascular diseases, pulmonary fibrosis, Ehler-danlos syndrome, Marfan syndrome, osteogenesis imperfecta, esophageal neoplasm, osteosarcoma, and endometriosis. Beyond that, this network showed the hub genes with higher betweenness, such as *hgf*, metallothionein 2a (*mt2a*), *mmp2*, fibrillin-1 (*fbn1*), *col1a1*, and *col1a2* could not only play a role in several diseases but also cause metastasis^38–40^. Moreover, it showed that survival hub genes, such as *fn1* and *serpine1*, could be involved in several diseases. The genes acting in the components of the extracellular matrix (ECM) pathway and HPV infection were also identified in this network (Fig. 5).

### Survival-correlation weighted network analysis

Interestingly, the agonist target network and the antagonist target network were separated, meaning that targeting one of the genes in each network did not have a significant impact on other networks. This can potentially reduce the chance of drug side effects (Fig. 4C). Based on this network, the antagonist targets mostly showed involvement in the ECM-receptor interaction and focal adhesion, while the agonist targets represented participation in the cell cycle pathway.

### Drug discovery

The genes with a negative correlation with better survival rates were searched in the Targetmine database to identify their antagonists. Some of the genes included *fn1,* K^+^/H^+^ ATPase transporting subunit alpha (*atp4a*), *serpine1*, *ctsk*, *pdgfrb*, and *kit.* There were also some genes that needed to be overexpressed, probably through using an agonist. Some of these genes were *cep55*, *cenpe*, *ect2,* anillin (*anln*), and checkpoint kinase 1 (*chek1*). Moreover, some candidate drugs for repositioning were found. Some of them were sorafenib, fostamatinib, troglitazone, quercetin (a natural flavonol found in vegetables preventing cancer and inflammation), and pantoprazole (Table 1).

**Table 1:** Drugs and compounds targeting survival genes and their correlated targets derived from Targetmine.

DrugBank interaction protein name	Action Type	DrugBank interaction compound identifier	DrugBank interaction compound name
Tumor necrosis factor ligand superfamily member 11	Inhibitor	Drug Bank: DB00480	Lenalidomide
Urokinase-type plasminogen activator	Inhibitor	DrugBank: DB00594	Amiloride
Fibrinogen alpha chain	Antagonist	DrugBank: DB00364	Sucralfate
Fibrinogen alpha chain	Antagonist	KEGG DRUG: D00446	Sucralfate
Fibronectin	Cleavage	DrugBank: DB08888	Ocriplasmin
Plasminogen activator inhibitor 1	Antagonist	DrugBank: DB00197	Troglitazone
Platelet-derived growth factor receptor beta	Antagonist	DrugBank: DB00619	Imatinib
Platelet-derived growth factor receptor beta	Inhibitor	DrugBank: DB12010	Fostamatinib
Mast/stem cell growth factor receptor Kit	Antagonist	DrugBank: DB00619	Imatinib
Mast/stem cell growth factor receptor Kit	Inhibitor	DrugBank: DB12010	Fostamatinib
CCAAT/enhancer-binding protein beta	Inhibitor	DrugBank: DB04216	Quercetin
Potassium-transporting ATPase alpha chain 1	Inhibitor	DrugBank: DB00213	Pantoprazole
Potassium-transporting ATPase alpha chain 1	Inhibitor	DrugBank: DB00338	Omeprazole
Potassium-transporting ATPase alpha chain 1	Inhibitor	DrugBank: DB00448	Lansoprazole
Potassium-transporting ATPase alpha chain 1	Inhibitor	DrugBank: DB00736	Esomeprazole
Potassium-transporting ATPase alpha chain 1	Inhibitor	DrugBank: DB01129	Rabeprazole
Potassium-transporting ATPase alpha chain 1	Inhibitor	DrugBank: DB05351	Dexlansoprazole
Proteinase-activated receptor 1	Antagonist	DrugBank: DB09030	Vorapaxar
Cathepsin K	Inhibitor	DrugBank: DB06670	Odanacatib

## Discussion

Recent studies showed that not only genetic factors but also some non-genetic factors contribute to gastric cancer^41^. Some pathways, including cell cycle*,* WNT/β-catenin signaling, focal adhesion, and nucleotide excision repair, are among the most important pathways in gastric cancer^42^. Currently, the main way of treating gastric cancer is still surgery, which might be followed by adjuvant therapy. However, surgery and adjuvant chemotherapy, as the gold standard therapy in gastric cancer, can increase median survival by only seven months^43^. Hence, understanding tumor biological pathways can be useful in finding new drugs and therapeutic methods for gastric cancer. In this study, using different bioinformatics tools and databases, the gene expression data in gastric cancer was analyzed and compared to a normal condition to identify the genes that are upregulated or downregulated in gastric cancer. The focus was given to survival genes, and the potential compounds that can affect patients’ survival were detected by performed analyses. To further understand pathological mechanisms of gastric cancer development, non-coding RNA interactions, viral causes, and pathways were also analyzed.

Cell cycle, which regulates cell proliferation and tumor growth, is one of the key pathways in all cancers including gastric cancer. According to our analysis, several genes including *cdc20, ccnb1, chek1, and mad2l1* participate in the cell cycle pathway. This pathway interacts with most pathways in gastric cancer, as discussed in the following.

**Figure 6:**
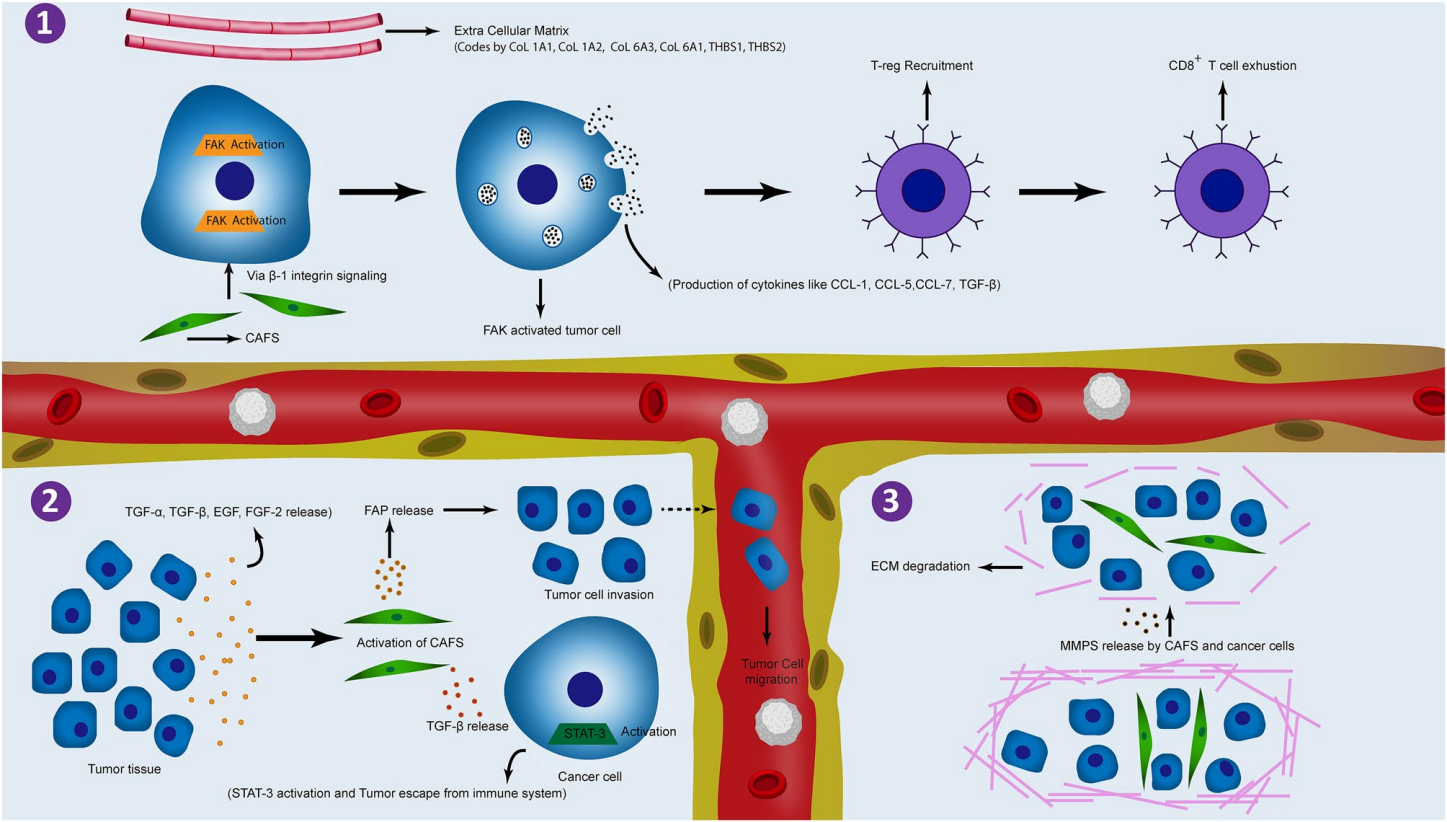
Focal adhesion signaling. (**1**) ECM and CAFs activating focal FAK in cancer cells leading to cytokine production. These cytokines can finally inhibit CD^8+^ T-cell activation. (**2**) Tumor cells release different cytokines resulting in CAF activation. This can finally cause tumor metastasis and escaping from immune cells induced by activated CAFs. (**3**) Activated CAFs and tumor cells can release MMPs leading to ECM degradation, which promotes tumor metastasis. *ECM: extracellular matrix; CAFs: cancer associated fibroblasts; FAK: focal adhesion kinase.

Based on our analysis, focal adhesion is a promising pathway, which was found to be upregulated in our analysis. It participates in multiple activities in tumor microenvironment (TME), immunosuppression, and metastasis. According to our analysis, *col1a1, col1a2, col6a3, col6a1, thbs1,* and *thbs2* are the main hub genes of this pathway, which mostly produce ECM and have an initial role in the activation of focal adhesion pathway^30–32^. Focal adhesion kinase (FAK) is an important part of the focal adhesion pathway, whose activation by these ECM compounds leads to an increase in cancer cell migration and survival, angiogenesis, cytokine production, and abnormal ECM accumulation^44^. Some of these cytokines and chemokines, namely CCL1, CCL5, CCL7, CXCL10, and TGF-β2. are responsible for T-reg cell recruitment, leading to CD^8+^ (cytotoxic T cell) exhaustion, which results in the reduction of tumoricidal cell function and helps cancer cells to escape from the immune system^45^. On the other hand, FAK activation can be induced by cancer-associated fibroblasts (CAFs) via β1 integrin, which can lead to proliferation, migration, and invasion potential in gastric cancer cells^46^. CAFs are crucial cells in tumor formation process. They can promote tumor cell growth via the promotion of cancer stemness or prevention of cancer cell recognition by T-cells. They are activated by pro-inflammatory cytokines such as TGF-α, TGF-β, FGF-2, and EGF, which are mainly secreted from tumor cells. Additionally, CAFs can release TGF-β leading to activation of STAT3 signaling pathway in tumor cells. CAFs and tumor cells can also secrete proteases, including matrix metalloproteinase, resulting in the breakdown of the cell’s basement membrane, which has a key role in cancer metastasis^47^. CAFs can also activate *fap* transcription, which was found in our analysis as a hub gene in the antagonist network (Fig. 4C). Since CAFs are overexpressed in the gastric cancer tissue and have a crucial role in tumor cell migration and invasion^48^, it can be concluded that focal adhesion and CAFs can be activated via different pathways, and they play key roles in cancer in different ways (Fig. 6).

The current study found that platelet activation is an important pathway in gastric cancer. *Col1a1*, *fga*, *fgg*, *f2r*, *col3a1*, and *col1a2* are the important genes of this pathway, which are upregulated in gastric cancer. According to KEGG, collagen fibers (*col1a1, col3a1, col1a2*) can activate GPVI, which leads to the initiation of platelet activation^30–32^.

**Figure 7:**
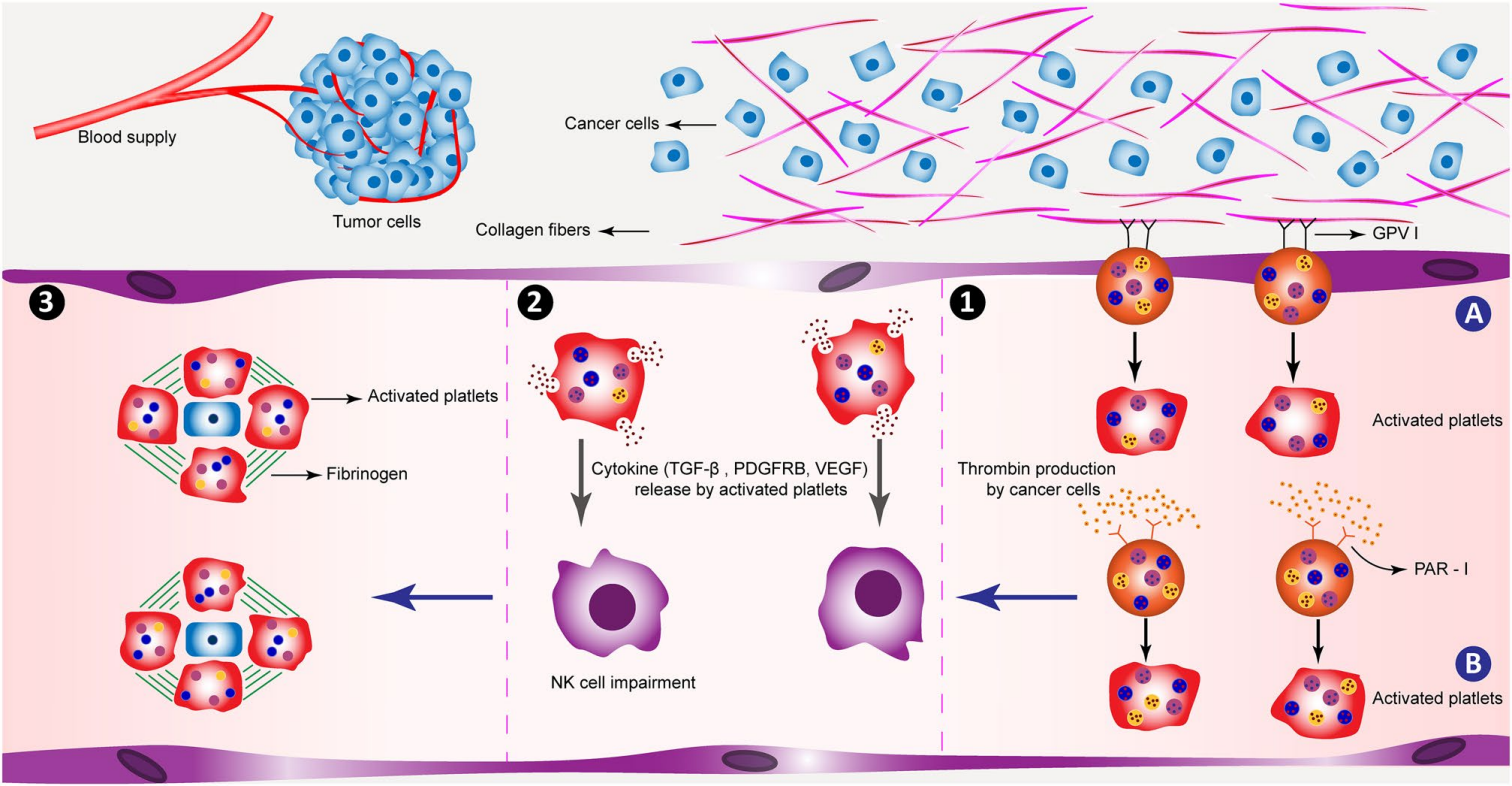
Platelet activation signaling pathway. (**1**) Platelets can be activated via two ways: (**A**) The GPVI receptor on platelets’ surface binds to collagen fibers in the ECM of tumor tissue; (**B**) Platelets can also be activated through binding to thrombin, which is produced by cancer cells via PAR-1 receptors on the platelets’ surface. Activated platelets can promote tumor metastasis via different ways. (**2**) They can also release different cytokines that impair NK cell’s function. These cytokines also can help tumor cell growth and vascularization. (**3**) They can cover up metastatic cells in collaboration with fibrinogen fibers helping metastatic cells to escape from the immune system. *NK: natural killer; GPVI: glycoprotein VI.

Thrombin is another molecule with multiple functions, which can not only attach to its receptor (PAR 1, coded by *f2r* gene) and activate platelets but also support angiogenesis by activating VEGF. Likewise, it could activate fibrinogen, which may cause cell proliferation and tumor growth in collaboration with PAR 1^49^. Platelet activation can help cancer development via different mechanisms. It may result in the release of transforming growth factor beta (TGF-β), VEGF, and platelet-derived growth factor (PDGF), which can cause tumor cell growth, angiogenesis, and neovascularization. Platelet and fibrinogen could coat tumor cells in the vascular and lymphatic systems, thereby supporting the escape of tumor cells from natural killer (NK) cells^49,50^. Platelets release TGF-β leads to the impairment of NK cell function^50^. This may imply that these mechanisms can hide tumor cells from the immune system and help them to metastasize into other organs and tissues (Fig. 7).

Another finding is that platelet activation by tumor tissue can cause a hypercoagulable state; consequently, patients have a higher risk of thrombotic conditions, namely deep vein thrombosis (DVT)^51^. Therefore, aspirin, as an anti-thrombotic agent, could be used in targeting platelet activation via acetylation of cyclooxygenase (COX)^52^. COX enzyme has two isoforms (COX1 and COX2), which are both inhibited by aspirin. COX1 inhibition leads to the anti-thrombotic effect of aspirin, while aspirin inhibition on COX2 enzyme results in anti-inflammatory effects. Its inhibitory effect on COX2 is 200 folds less potent than COX1^52^. The results of this study suggest that aspirin could act as a potential therapeutic agent in cancer. According to recent studies, the long-term use of aspirin was associated with a reduced risk of having gastric cancer, especially non-cardiac type gastric cancer^53,54^. In addition, aspirin could inhibit the growth of gastric cancer cell lines via suppressing the survivin protein and induction of apoptosis^55,56^. Thus, although aspirin is associated with stomach mucosal damage^57^, it might prevent gastric cancer occurrence via inhibition of platelet activation and survivin, as well as apoptosis induction.

It was also found that upregulation of the HPV infection pathway plays an important role in gastric adenocarcinoma. HPV is one of the most important carcinogenic viruses in humans. Different HPV serotypes have been discovered, among which HPV-16 and HPV-18 are the most prevalent mucosal high-risk serotypes, associated with nearly 90% of human cervical cancers and 20% of oral cancers^58–60^. The *E6* and *E7* genes of HPV play a causative role in cancer progression; the former promotes degradation of *p53* through its interaction with *E6AP*, an *E3* ubiquitin ligase, while the latter binds to the retinoblastoma protein (*prb*) and disrupts its complex formation with *E2F* transcription factors so that *p53* is suppressed and *rb* is promoted causing a higher rate of cell division^61^. According to our study, *pdgfrb*, which is a mitogen for the cells with a mesenchymal origin, is an important survival gene whose underexpression is associated with better outcomes in gastric cancer patients. On the other side, according to VIRHOSTNET 3.0, available at https://virhostnet.prabi.fr/, the E6 protein of HPV can directly induce *pdgfrb* gene expression^62^.

The result of our study is consistent with a meta-analysis of 30 studies, including 1,917 gastric cancer patients and 576 controls. It was revealed that HPV prevalence was 28% higher in patients with gastric cancer, which was significant compared to the control group. It was found that HPV-positive patients with gastric cancer were higher in the Chinese than non-Chinese population, which may be the reason for the higher prevalence of gastric cancer in China^63^. Interestingly, the microarray database used in this study was prepared from people of Hangzhou in China^25^. Therefore, it is hypothesized that HPV may be a risk factor for gastric adenocarcinoma due to its direct effect on survival genes, as it is associated with a higher risk of cervical, colorectal, esophageal, and oropharyngeal cancers^64^. Nevertheless, there are other studies against our findings, which did not detect HPV in the gastric cancer samples^65^. Hence, more studies in various countries are needed to verify this hypothesis.

### Survival and drug analysis

The results indicated that gastric acid secretion, which is a crucial pathway to digest food in normal stomach physiology, plays a key role in gastric adenocarcinoma progression. *Atp4a* had a strong positive linear correlation with *ghrl* (among survival genes), and both of them were underexpressed in gastric cancer according to GEPIA boxplot, which resulted in gastric acid secretion downregulation. Therefore, *ghrl* and possibly *atp4a* have a protective role in gastric cancer.

In our study, PPI drugs were shown to target the *atp4a* gene, so they might help patients to have a better prognosis. However, some studies showed PPIs as a risk factor in developing gastric cancer^66^. According to these contrary results, we decided to study the mechanism of gastric acid secretion to gain a better insight into PPIs’ effect on *atp4a* and ghrelin interaction. Since a few studies have shown the relationship between *atp4a* and ghrelin, we were not certain if *atp4a* expression was the result of ghrelin expression. On the other hand, gastrin can stimulate *atp4a*, which can code for potassium hydrogen ATPase channels and increase acid secretion, as an important risk factor for gastric cancer^67^.

**Figure 8:**
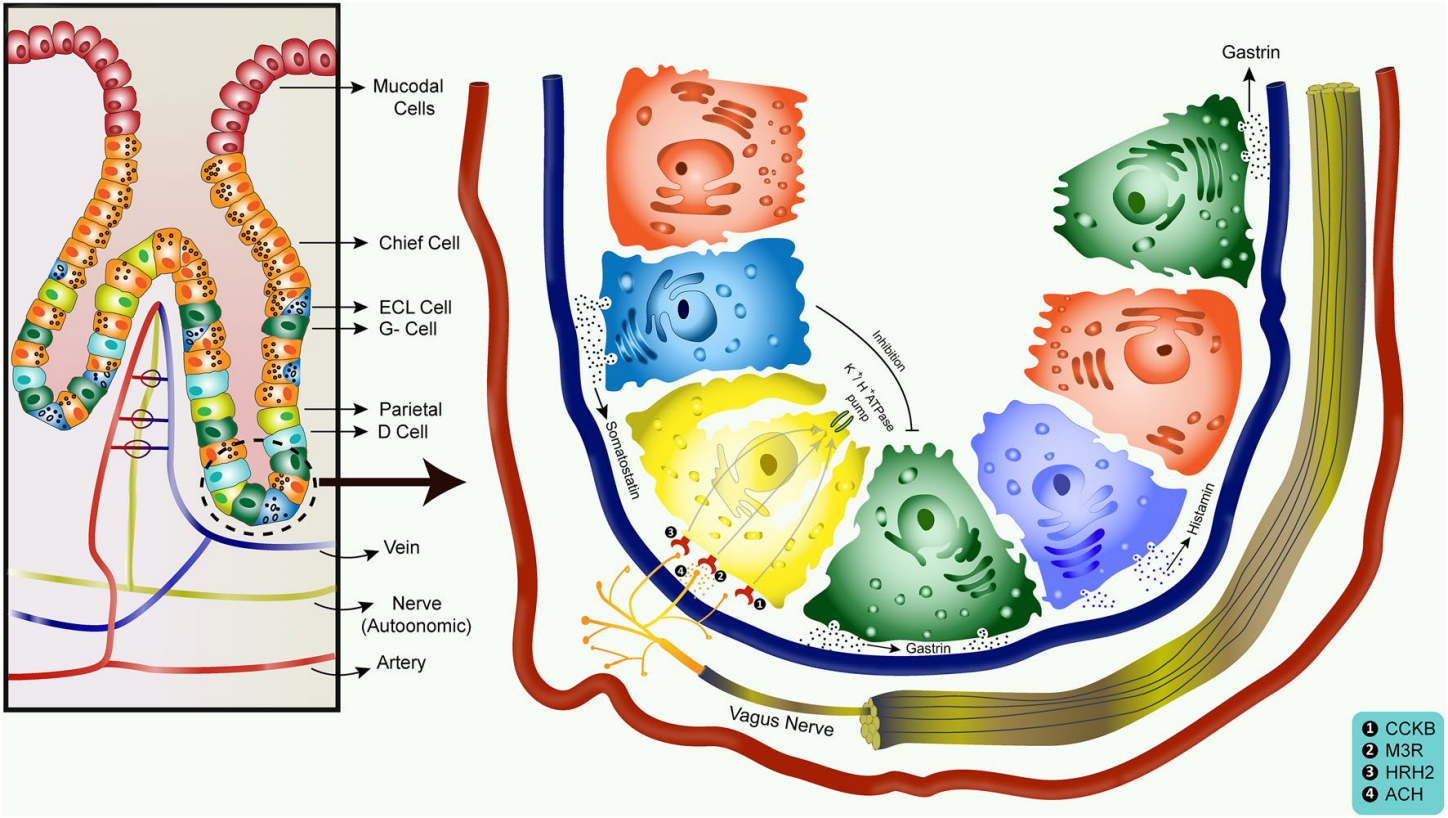
Gastric acid secretion signaling pathway. Gastrin is mainly produced by G- cells that induce CCKβR function. It can result in gastric cancer progression and prompts acid formation in parietal cells. Acetylcholine (ACH) released from the vagus nerve ending binds to Muscarinic M_3_ receptors (M3R). Histamine produced by ECL cells binds to HRH2. All these together result in the activation of *atp4a* gene, which codes K^+^/H^+^ ATPase pumps and increases acid secretion. D-cells release somatostatin, which has an inhibitory effect on G-cells finally resulting in reducing the acid secretion. *HRH2: histamine receptor H2; ECL: enterochromafin cells.

Gastrin hormone induces tumor cell growth, migration, autophagy, and survival^68^, and its secretion by G-cells is inhibited by somatostatin (SST) release through D cells^69^. Gastrin also can stimulate cholecystokinin β receptors (CCKβR), leading to the growth of gastric cancer tumor cells^70^. Thus, it is hypothesized that a lower level of SST leads to a higher level of gastrin, which increases gastric cancer occurrence. Downregulation of SST was also found in the DEGs of our study. Based on the aforementioned findings, SST decrease and gastrin increase have a synergistic effect on gastric cancer development, while ATP4A and GHRL decrease act oppositely so that their net effect on gastric cancer progression depends on the influences of each axis of gastric cancer development. Based on the results of our study, gastrin upregulation can also increase histamine release, and the downregulation of SST has the same effect. The result may imply that the underexpression of *ghrl* gene possibly affects enterochromafin cells (ECL) through decreasing histamine release; the effect of *ghrl* on histamine is contrary to gastrin and somatostatin^71^. Histamine can act through histamine receptor h2 (*hrh2*), which plays a stimulatory effect on acid secretion^72^. Based on GEPIA, *hrh2*, the same as *ghrl*, is a survival gene in gastric cancer whose underexpression is associated with better survival in patients (downregulated based on our DEGs) (Fig. 8). These data suggest the same mechanisms of gastric cell protective response against cancer development.

Based on these findings, PPIs may have therapeutic effects on gastric cancer. According to Cheung and Leung, the long-term use of PPIs could increase the risk of gastric cancer by nearly twofold^66^, while in other studies investigating PPI association with gastric cancer cell lines, rabeprazole, a second-generation PPI drug, could reduce gastric cancer cell survival in vitro^73^. The probable interpretation could be that although the long-term use of PPIs can lead to higher rates of gastric cancer in the normal population, PPI drugs could reduce cancer progression in patients diagnosed with gastric adenocarcinoma.

According to our analysis, *pdgfrb* is mostly involved in pathways such as focal adhesion, PI3K-AKT signaling pathway, the pathways in cancer, and HPV infection. Meanwhile, *kit* mostly participates in PI3K-AKT signaling pathway and the pathways in cancer, and both *pdgfrb* and *kit* are survival genes whose overexpression leads to poor survival for patients. Fostamatinib, which is used for rheumatoid arthritis and immune thrombocytopenic purpura (ITP) treatment^74,75^, can also be used as a potential inhibitor of *pdgfrb* and *kit*^76^. Thus, fostamatinib may be a probable small molecule for treating gastric cancer to reduce cell proliferation, metastasis, and cancer progression. A recent clinical trial for fostamatinib performed on multiple malignancies (advanced colorectal, non-small cell lung, head and neck, thyroid, and renal cell carcinomas, as well as pheochromocytoma) showed its anti-tumor activity in several patients^77^. However, its effect on gastric adenocarcinoma has not been studied yet.

Fibrinolytic system can also be a potential target for cancer therapy. Plasmin is the key compound of this system, which is derived from plasminogen. Plasminogen is an inactive enzyme, which is converted to plasmin by tissue- or urokinase-type plasminogen activators (tPA or uPA). uPA is coded by *plau* gene. Fibrinolytic system takes part in cancer in different ways. Plasmin can activate MMPs, which have a crucial role in ECM degradation, cancer cell migration, and metastasis. The uPA system can induce cell proliferation via increasing growth factors such as VEGF, EGF, FGF2, and TGF-β. It also causes a reduction in apoptosis, leading to cancer cell immortality^78,79^. Hence, using uPA inhibitors could be useful in cancer treatment. Amiloride is a selective uPA inhibitor commonly used for inhibiting sodium reabsorption through sodium channels in the renal epithelial cells^80^. It can reduce gastric cancer peritoneal metastasis, which could be useful at the end stages of gastric cancer^81^. The *serpine1* gene codes plasminogen activator inhibitor 1 (PAI-1). Thus, PAI-1 overexpression, as the inhibitor of uPA system, must have a positive effect on patients’ survival by the inhibition of cell proliferation, metastasis, and apoptosis induction^79^. However, in our analysis, we found that *plau* has a positive linear correlation with *serpine1*, so inhibiting both of them may have a synergistic effect in gastric cancer treatment. The PAI-1 level is higher in gastric cancer tissue than in normal tissue, and its overexpression leads to poor survival in gastric cancer patients via increasing tumorigenicity and inhibition of cancer cells apoptosis^82–84^, as was confirmed by GEPIA. Therefore, using a PAI-1antagonist as well as uPA antagonists, can be useful in gastric cancer treatment. Troglitazone, a PAI-1inhibitor, was once used as an anti-hyperglycemic agent, but it was withdrawn in the year 2000 because of its hepatotoxicity. Thus, finding a way to reduce its toxicity can candidate it as a potential drug for gastric cancer^80^.

### MicroRNA analysis

MicroRNAs are small non-coding RNAs with important effects on gene regulation. Their regulation is being interrupted in a variety of cancers. Many different mechanisms are involved in microRNA dysregulation, such as amplification or deletion of microRNA genes, epigenetic changes and defects in the microRNA biogenesis machinery, and abnormal transcriptional control of microRNAs. They also have a considerable role in cancers by regulating cell proliferation signaling pathways, cell resistance to apoptosis, invasion, metastasis, and inactivating of tumor suppressor mechanisms, which help tumor cells to grow and build a tumor mass. They also affect the clinical features and therapeutic outcomes of tumors, which could be sensitive and specific biomarkers for diagnostic processes and therapeutic goals^85^. MicroRNAs also have a key role in gastric cancer. According to our studies, MALAT1 is a hub microRNA among others in gastric cancer (Fig. 4B). According to recent studies, MALAT1 function varies in different tumors, which may act as a tumor suppressor gene or have an oncogenic effect on different cancers. MALAT1 directly binds to SOX2 mRNA, which enhances its stability so that it can have a positive effect on the regulation of stemness of gastric cancer cells^86^. Likewise, MALAT1 can be a powerful candidate for prognostic goals in gastric cancer. Some studies reveal that MALAT1 level is higher in patients with a distant gastric cancer metastasis than those without distant metastasis in the control group^87^. As discussed above, the role of microRNAs in different cancers is proved^88^, but still more investigations are needed to reveal other dimensions of their effect on cancers. They can be very good biomarkers to solve challenges in finding specific and sensitive biomarkers for different types of cancers.

### Gene disease network analysis

Gastric cancer is a complicated disease, and different pathways take part in its pathogenesis. Gastric cancer can lead to secondary metastatic cancers in other tissues. According to our analysis, different diseases and syndromes are associated with gastric cancer, such as liver carcinoma, malignant neoplasia of the prostate, atherosclerosis, liver fibrosis, myocardial infarction (MI), and lung carcinoma. Among these diseases, liver carcinoma, gastric cancer, and lung carcinoma are correlated via the baculoviral inhibitor of apoptosis repeat-containing 5 (*birc5*) gene. *Birc5* is a member of the inhibitor of apoptosis (IAP) gene family, which codes proteins that can prevent cell apoptosis^89^. According to GEPIA, *birc5* is overexpressed in these three cancers^36^, so that it can lead to the activation of the anti-apoptotic pathway, which is a crucial pathway in cancer progression. Gastric cancer and atherosclerosis are connected to each other via the *serpine1* gene. On the other hand, gastric cancer and MI are similar in *plau* gene. As discussed above, these two genes participate in the complement coagulation pathway. In fact, the complement coagulation pathway is an interplay between the complement system as a part of innate immunity and the coagulation pathway. Inflammation is one of the key factors that can activate the complement coagulation pathway^90^. In both MI and atherosclerosis, inflammation is an important part of the pathogenesis, so that this pathway can interconnect MI and atherosclerosis with gastric cancer. The malignant neoplasias of prostate and gastric cancers also share many genes, one of the most important of which is *mt2a*. The *mt2a* gene is a member of the metallothionein family of genes, which code proteins that have a substantial role in the hemostatic control of metals in cells and detoxification of heavy metals influences apoptotic and autophagy pathways^89^. According to GEPIA, *mt2a* is underexpressed in both of these cancers^36^, which leads to less detoxification of heavy metals and increased risk of these cancers in the same way.

These data show an interesting correlation between gastric cancer and other diseases, which can help in predicting other diseases induced by gastric cancer tumor or other diseases with a predisposing role in gastric cancer.

### Limitations and perspectives for future studies

1- Although the microarray dataset was paired data, and its PCA graph was well separated, it was not mentioned which part of the stomach was used for collecting the tumor samples.

2- For a better analysis of each type of cancer, it is better to have samples that are collected from the same disease stage. However, it was not mentioned in the source of microarray data.

3- In this study, all the linear relationships that existed between gene expression were used. Further inclusion of nonlinear relationships in future studies might generate more accurate data on the relationship between proteins, and probably more drugs could be suggested.

4- Using RNA-Seq technology to measure gene expression might lead to more reliable results. However, due to the unavailability of paired RNA-Seq data in this study, paired microarray data was used, which matched better and could produce more valid results. Besides, to increase accuracy in this study, key genes with significant differential expression in both microarray dataset and TCGA data (analyzed by GEPIA) were employed for drug target discovery.

## Conclusion

Totally based on a multilevel systems biology analysis, the hub genes in gastric adenocarcinoma showed participation in the pathways such as focal adhesion, platelet activation, gastric acid secretion, HPV infection, and cell cycle. In the survival and drug analysis, fostamatinib and troglitazone were found as potential drugs targeting survival and hub genes in gastric adenocarcinoma. The fibrinolytic system and gastric acid secretion are two important pathways for drug analysis. PPIs are hypothesized to have a therapeutic effect on patients with gastric cancer, but their long-term administration can induce cancer in the normal population. Although microRNA analysis showed the potential role of MALAT1 in gastric cancer pathogenesis, there are presently few studies available on this subject. More future studies may help in finding novel therapies or new biomarkers.

Moreover, gene-disease network showed many mechanisms and genes being shared in gastric cancer and other diseases. Therefore, a drug or therapeutic approach might be useful for more than one of these diseases due to their similar pathways. Through these analyses, a new window is opened to achieve a better understanding of gastric cancer as one of the most complicated tumors. Further research in this field can deepen our knowledge and drive advancement in developing novel therapeutic approaches for gastric cancer as well as some other related diseases.

## Supplementary Information

Supplementary Information.

## Data Availability

The dataset used in this study was obtained from a publicly available repository, gene expression omnibus (GEO) at https://www.ncbi.nlm.nih.gov/geo/. All primary expression files are deposited in the CEL files.
