# Supplementary material for: Identification of hub pathways and drug candidates in gastric cancer through systems biology
Source: Sci Rep. 2022 Jun 1;12:9099. doi: 10.1038/s41598-022-13052-0 (PMC9160265; doi:10.1038/s41598-022-13052-0)
Supplement: Supplementary file 1 — Supplementary Information. [file 41598_2022_13052_MOESM1_ESM.docx]

**Table S1:** The microarray data of tumor and normal samples

| Sample name | Sample type |
| --- | --- |
| GSM2109532 | Tumor |
| GSM2109533 | Normal |
| GSM2109534 | Tumor |
| GSM2109535 | Normal |
| GSM2109536 | Tumor |
| GSM2109537 | Normal |
| GSM2109538 | Tumor |
| GSM2109539 | Normal |
| GSM2109540 | Tumor |
| GSM2109541 | Normal |
| GSM2109542 | Tumor |
| GSM2109543 | Normal |
| GSM2109544 | Tumor |
| GSM2109545 | Normal |
| GSM2109546 | Tumor |
| GSM2109547 | Normal |
| GSM2109548 | Tumor/ was removed |
| GSM2109549 | Normal/ was removed |
| GSM2109550 | Tumor |
| GSM2109551 | Normal |

**Table S2** The pathways of regulated and upregualted genes obtained through enrichment analysis by GO. Note: Regulated genes are shown as “Top of list”; while upregualted genes are shown as “Bottom of list.

| # Term ID | Term description | Enrichment score | Direction | False discovery rate | Method |
| --- | --- | --- | --- | --- | --- |
| GO:0000278 | Mitotic cell cycle | 2.37203 | Bottom of list | 8.25E-12 | ks |
| GO:0022402 | Cell cycle process | 2.28277 | Bottom of list | 8.94E-12 | ks |
| GO:0007049 | Cell cycle | 2.22176 | Bottom of list | 9.17E-12 | ks |
| GO:1903047 | Mitotic cell cycle process | 2.37646 | Bottom of list | 2.67E-11 | ks |
| GO:0051301 | Cell division | 2.28014 | Bottom of list | 1.04E-09 | ks |
| GO:0043062 | Extracellular structure organization | 2.49243 | Bottom of list | 1.79E-06 | ks |
| GO:0010564 | Regulation of cell cycle process | 2.2449 | Bottom of list | 4.49E-06 | ks |
| GO:0030198 | Extracellular matrix organization | 2.52088 | Bottom of list | 4.91E-06 | ks |
| GO:0001568 | Blood vessel development | 2.04289 | Bottom of list | 0.0001 | ks |
| GO:0001944 | Vasculature development | 2.04614 | Bottom of list | 0.00015 | ks |
| GO:0034220 | Ion transmembrane transport | 0.00874716 | Top of list | 0.0032 | ks |
| GO:0007586 | Digestion | 0.747112 | Top of list | 0.0035 | ks |
| GO:0048514 | Blood vessel morphogenesis | 1.93191 | Bottom of list | 0.0035 | ks |
| GO:0000226 | Microtubule cytoskeleton organization | 1.93161 | Bottom of list | 0.0039 | ks |
| GO:0070925 | Organelle assembly | 2.21829 | Bottom of list | 0.0074 | ks |
| GO:0000075 | Cell cycle checkpoint | 2.27006 | Bottom of list | 0.0076 | ks |
| GO:0045787 | Positive regulation of cell cycle | 2.16313 | Bottom of list | 0.0096 | ks |
| GO:0006811 | Ion transport | 0.0463797 | Top of list | 0.0115 | ks |
| GO:0007155 | Cell adhesion | 1.74669 | Bottom of list | 0.0152 | ks |
| GO:0007010 | Cytoskeleton organization | 1.26456 | Bottom of list | 0.016 | ks |
| GO:0030177 | Positive regulation of Wnt signaling pathway | 2.73238 | Bottom of list | 0.0161 | afc |
| GO:0030199 | Collagen fibril organization | 2.69326 | Bottom of list | 0.0179 | afc |

**Table S3** The first 30 hub genes and their centrality parameters including degree, betweenness, closeness, eigen centrality with their modularity class value.

| ID | Degree | Betweenness centrality | Modularity class | Eigen centrality |
| --- | --- | --- | --- | --- |
| FN1 | 59 | 1437.787182 | 0 | 1 |
| MKI67 | 27 | 436.496776 | 3 | 0.41507 |
| SPP1 | 38 | 410.415195 | 0 | 0.726392 |
| MMP2 | 39 | 366.231726 | 2 | 0.800203 |
| CCNB1 | 26 | 279.991548 | 3 | 0.392646 |
| KIT | 16 | 265.744379 | 1 | 0.23902 |
| SOX2 | 20 | 228.048141 | 1 | 0.273141 |
| SERPINE1 | 29 | 208.677119 | 0 | 0.583734 |
| KLF4 | 15 | 184.917453 | 1 | 0.150298 |
| BIRC5 | 22 | 178.98089 | 3 | 0.369863 |
| PRKACB | 7 | 155.109392 | 1 | 0.085652 |
| COL1A1 | 39 | 151.637101 | 2 | 0.804569 |
| CEP55 | 22 | 144.894865 | 3 | 0.358638 |
| PLAU | 17 | 138.92431 | 0 | 0.329287 |
| MGAM | 5 | 136.266734 | 0 | 0.061044 |
| BMP2 | 22 | 135.713954 | 1 | 0.431182 |
| THBS1 | 31 | 127.081726 | 2 | 0.699943 |
| UBE2C | 26 | 125.907413 | 3 | 0.349822 |
| WNT5A | 14 | 118.152492 | 1 | 0.237726 |
| COL1A2 | 37 | 113.887074 | 2 | 0.789705 |
| MSH2 | 12 | 112.805236 | 3 | 0.213986 |
| TIMP2 | 19 | 108.587554 | 2 | 0.464034 |
| TIMP1 | 35 | 106.128144 | 0 | 0.732976 |
| CHGA | 8 | 102.451179 | 1 | 0.048114 |
| FBN1 | 29 | 102.189664 | 2 | 0.66602 |
| THY1 | 19 | 99.527009 | 1 | 0.403218 |
| THBS2 | 26 | 93.184586 | 2 | 0.63253 |
| KPNA2 | 22 | 91.831071 | 3 | 0.322768 |
| CYP3A4 | 8 | 89.288492 | 1 | 0.049635 |
